# When Multiple Conditions Converge: An Unusual Case of Infectious Aortitis in a Patient With Prurigo Nodularis and an Abdominal Aortic Aneurysm Endograft

**DOI:** 10.7759/cureus.48519

**Published:** 2023-11-08

**Authors:** Drew Thompson, Larissa Check, Chris Pun, Connor Puett, Mohamed Faris

**Affiliations:** 1 Internal Medicine, Grand Strand Medical Center, Myrtle Beach, USA

**Keywords:** methicillin-resistant staphylococcus aureus, prurigo nodularis, tangier's, gram-negative bacteremia, infectious aortitis

## Abstract

Infectious aortitis is a rare disease process that presents with mortality varying from 60% to 90%, even with aggressive treatment. This is a case involving a 69-year-old male who initially presented for acute encephalopathy. The patient’s past medical history included coronary disease status post coronary bypass graft, abdominal aortic aneurysm status post endograft repair, prurigo nodularis, Tangier's disease, type 2 diabetes mellitus, and stage 3b chronic kidney disease. Initially, the work-up was unrevealing for a cause of the patient’s acute encephalopathy. However, astute clinical evaluation led to the diagnosis of methicillin-sensitive *Staphylococcus aureus* (MSSA) bacteremia and abdominal infectious aortitis. Prurigo nodularis is a chronic dermatologic condition characterized by the development of intensely pruritic, firm nodules or bumps on the skin associated with itching and scratching. Prurigo nodularis itself does not directly result in bacteremia. However, in rare cases, severe and persistent scratching due to prurigo nodularis can lead to breaks in the skin, creating an entry point for bacteria to spread by the hematogenous route. Certainly, it is highly unusual to have a combination of prurigo nodularis, MSSA bacteremia, and abdominal aortic aneurysm endograft infection. Given the severity of these conditions individually, the combination presents a unique and challenging clinical scenario that requires prompt and coordinated management by a multidisciplinary team. This case report aims to provide new insights into the potential risk factors, clinical course, and management strategies for these combined conditions.

## Introduction

Infectious aortitis (IA) is a rare inflammatory process of the aorta due to the colonization and proliferation of bacteria, fungi, or viruses within the aortic wall [1]. This largely uncommon cause of aortitis can develop through a variety of the following etiologies: bacterial infection of an atherosclerotic plaque or an injury of the aortic intima [1], or it can also result from a septic embolus spreading through the vasa vasorum to the aortic intimal layer, bacterial seeding from an endovascular procedure, and direct bacterial infection related to trauma [1]. Prior to the widespread availability of antibiotics, syphilis, group A *Streptococcus*, *Streptococcus pneumoniae*, and *Haemophilus influenzae* were the most common causes of IA [1]. However recently, *Salmonella* species and *Staphylococcus aureus* are implicated in most cases of IA [2,3]. More specifically, *Salmonella* species is more prevalent in IA of the abdomen, and in developing countries, *Mycobacterium tuberculosis* causes two-thirds of aortitis cases [1]. IA is typically seen in ages over 50 years old within the American and European populations with incidences ranging from 1% to 2% [4]. Risk factors associated with the development of IA include male gender, age over 50 years, diabetes, syphilis, infective endocarditis, intravenous drug use, and pre-existing aortic abnormalities, such as atherosclerosis, prostheses, aneurysms, congenital vascular abnormalities, and cystic medial necrosis [2,5,6]. Chronic skin conditions such as prurigo nodularis are atypical risk factors for IA.

## Case presentation

**Figure 1:**
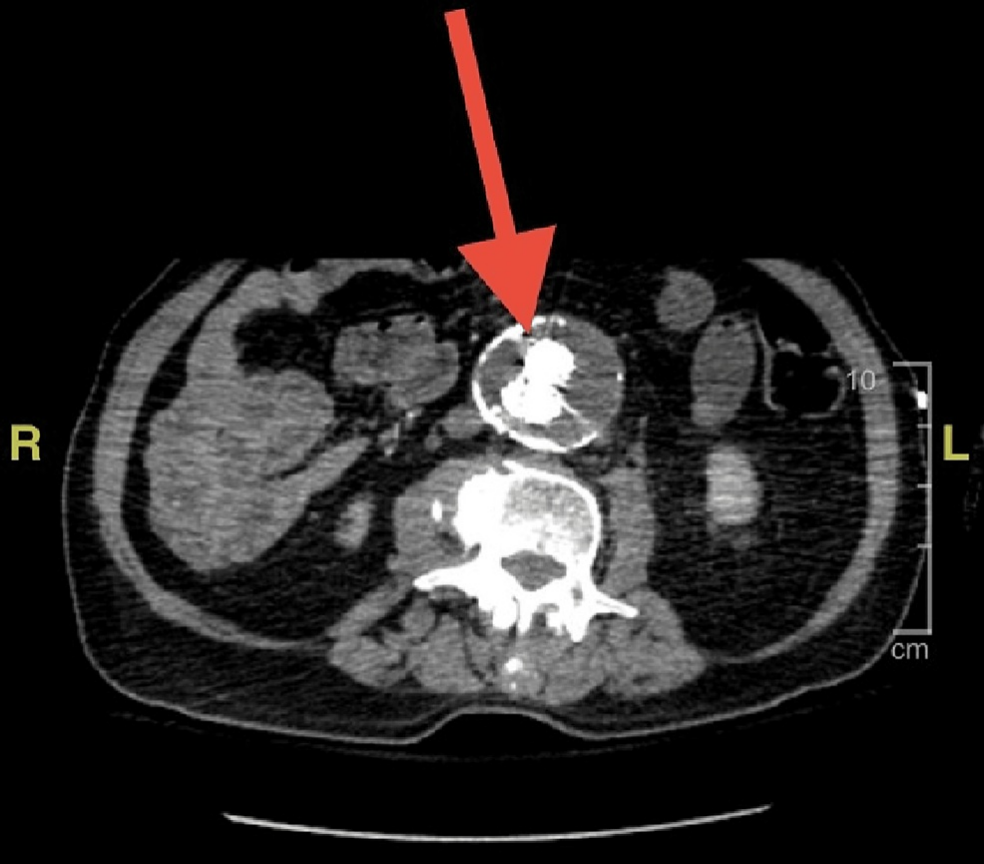
CT of the abdomen and pelvis with contrast on admission showing infrarenal abdominal aortic aneurysm with the aortobiiliac stent graft with the aneurysm sac measuring up to 4.6 x 5.5 cm in greatest dimension (red arrow). This size was not significantly changed when compared to imaging done in 2019.

This case involves a 69-year-old male with a past medical history significant for coronary artery disease status post four-vessel coronary artery bypass graft, abdominal aortic aneurysm status post endograft repair, prurigo nodularis, Tangier's disease, type 2 diabetes mellitus, and chronic kidney disease, who was admitted for acute encephalopathy. A full medication review was performed, and the patient was not on any immunosuppressant medications. The patient was reportedly found down by a neighbor with an unknown total downtime. Prior to this incident, the patient was independent and the neighbor reported no preceding changes in behavior patterns. On our physical exam, the patient was encephalopathic and was not oriented to the self, place, or time. The exam further revealed he was in sinus tachycardia, his abdomen was mildly tender in the right upper quadrant and epigastric region. He was also noted to be missing his entire nose due to a resection from chronic prurigo nodularis. On admission, computed tomography (CT) imaging of the head, chest, and abdomen/pelvis were completed in the emergency department and were unremarkable. The initial CT of the abdomen is shown below (Figure 1). Furthermore, the patient showed signs of systemic inflammatory response with a maximum temperature of 101.8° Fahrenheit, a heart rate ranging from 100s to 130s, and a white blood cell count of 22.8. Lactic acid on admission was elevated at 6.9 mmol/L. As a result, the patient had blood cultures drawn and empiric antibiotics were initiated to include intravenous vancomycin, cefepime, and clindamycin. Clindamycin was initiated due to concern for necrotizing fasciitis in the setting of non-healing lesions resulting from prurigo nodularis.

**Figure 2:**
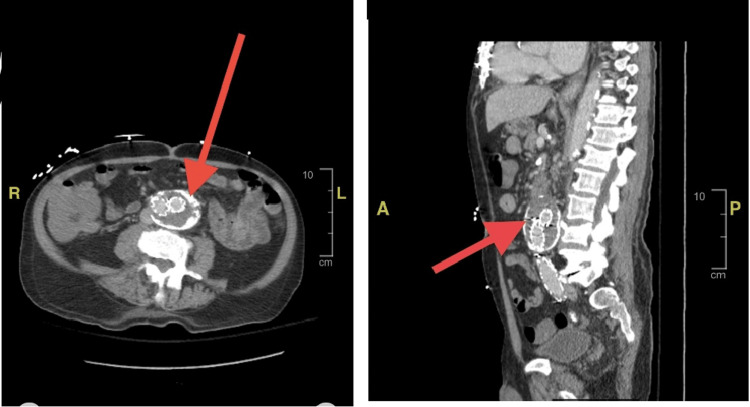
CT of the abdomen and pelvis with contrast on admission showing an infrarenal abdominal aortic aneurysm with the aortobiiliac stent graft now with inflammatory stranding in the proximal aspect of the graft and the aortic wall concerning for aortitis or an infected graft. The first image shows an axial view and the second image shows a coronal view. Both arrows point to the location with inflammatory changes.

The initial blood cultures showed methicillin-sensitive *Staphylococcus aureus* (MSSA) bacteremia in two out of two cultures. A repeat culture was ordered and all antibiotics were de-escalated to a single agent of intravenous cefazolin. It was thought that the bacteremia was seeded from the patient’s skin and soft tissue lesions. Repeat cultures were drawn every 48 hours and remained positive in three consecutive culture sets. The fourth culture returned negative. The patient at the time met one major Duke’s criteria for possible infectious endocarditis and thus a transthoracic echocardiogram was ordered. The echocardiogram was negative for intracardiac lesions or abscesses on the valve, septum, or papillary muscles. A transesophageal echocardiogram was subsequently ordered due to persistently positive blood cultures but was also unrevealing. On the sixth day of hospitalization, it was noted that the patient endorsed worsening abdominal tenderness on his physical exam. This physical exam finding prompted a repeat CT of the abdomen with intravenous contrast. The abdominal imaging resulted in new findings of inflammatory stranding and mural thickening involving the proximal abdominal aorta consistent with infectious aortitis likely involving the endograft (Figure 2).

Infectious disease was consulted in the setting of this complicated infection in addition to vascular surgery for possible surgical intervention. Vascular surgery assessed the patient and recommended treatment with intravenous antibiotics and a follow-up for possible surgical removal of the graft. Fortunately, the patient responded well to intravenous antibiotics and completed a 12-week course of intravenous cefazolin prior to discharge.

## Discussion

IA, also known as mycotic aortitis, is a rare infection that affects the aorta, which is the large artery that carries blood from the heart to the rest of the body [6]. This condition occurs when bacteria or fungi invade the walls of the aorta, causing inflammation and damage. It can lead to serious complications such as aneurysm, rupture, and sepsis if left untreated [6,7]. Timely diagnosis and treatment with antibiotics or antifungal medication are crucial to manage this condition. Prompt recognition of infectious abdominal aortitis is critical, as the untreated condition is considered universally fatal, secondary to sepsis or hemorrhage [8]. However, establishing this diagnosis can be difficult. Most commonly, the clinician is confronted with a febrile patient who shows signs of elevated inflammatory markers and leukocytosis. Although infection is commonly suspected, blood cultures are negative in one-third to one-half of cases and few patients present with abdominal pain or a pulsatile abdominal mass [1,6,7].

A high level of clinical suspicion is therefore required and should prompt further diagnostic evaluation in patients with known aortic disease, such as prominent atherosclerosis or abdominal aortic aneurysm (AAA) [7]. As in this case, a history of surgical AAA repair is of particular importance, as the incidence of aortic graft infection may approach 1%, and if present, defines a unique clinical and surgical setting [8]. Contrast-enhanced CT is the diagnostic study of choice [8]. It has a high sensitivity for aortic pathology and provides important information to guide management, such as the location and extent of vessel involvement as well as the presence of complications, including aneurysm, dissection, abscess, bleeding, and fistula presence [8,9]. Blood cultures and tissue cultures obtained at surgery have shown gram-negative bacteria and *Staphylococcus aureus* to be the most common bacterial culprits, underlying the current recommendations regarding empiric antibiotic choices pending guidance from cultures. *Salmonella* poses a unique risk, and aortitis should be considered in patients with a history of aortic disease and salmonellosis. Once the diagnosis of IA has been established and appropriate antibiotic treatment initiated, surgical intervention must be considered [9].

Surgery can greatly improve the chances of survival for IA. Without surgery, the survival rate is only around 10% with medical management alone [1]. However, with surgery, the survival rate increases to over 75% [1]. However, there are many clinical details that influence surgical outcomes, such as the presence of an aneurysm and whether endovascular repair is an option. As this case demonstrates, patients with atherosclerotic abdominal aortic disease, especially those who have required previous surgical intervention with grafting, are at a high risk of endograft infection. The prognosis of IA with an infected graft in this setting is poor. Attempts to minimize surgical risk using percutaneous abscess drainage or limited surgical debridement without graft removal have shown little success, and the mortality following a complete surgical debridement with graft removal is about 30% [8]. Antibiotic therapy is typically continued for at least six to 12 weeks after surgery [7] and is closely directed by the patient’s clinical course as well as inflammatory markers and blood culture monitoring [10].

## Conclusions

IA is a rare disease process that comes with extremely high mortality even with aggressive treatment. There is limited updated data involving necessary interventions of IA that include long-term antibiotic therapy, surgical intervention, or a combination of both. Initially, the imaging performed did not show signs of an infection, but when the imaging was repeated six days later, it did reveal that IA was present. Our leading hypothesis is that this patient’s chronic scalp lesions secondary to his prurigo nodularis created an entry point resulting in bacteremia that ultimately seeded his abdominal aortic endograft. In most cases, surgical intervention is necessary to control the bacteremia, but in this case, medical management alone resulted in a satisfactory resolution of the bacteremia. We treated the patient using intravenous antibiotics for 12 weeks and planned to closely monitor them with the assistance of vascular surgery. There remains a strong possibility that the aortic endograft may need to be removed in the future to provide definitive treatment.
